# Diagnostic Value of Ischemia-Modified Albumin in Acute Coronary Syndrome and Acute Ischemic Stroke

**Published:** 2013

**Authors:** Birsen Ertekin, Sedat Kocak, Zerrin Defne Dundar, Sadik Girisgin, Basar Cander, Mehmet Gul, Sibel Doseyici, Idris Mehmetoglu, Tahir Kemal Sahin

**Affiliations:** 1 Birsen Ertekin, Department of Emergency Medicine, Beyhekim State Hospital, Konya, Turkey.; 2 Sedat Kocak, Department of Emergency Medicine, Meram Medical Faculty, Konya University, Konya, Turkey.; 3 Zerrin Defne Dundar, Department of Emergency Medicine, Konya Research and Training Hospital, Konya, Turkey.; 4 Sadik Girisgin, Department of Emergency Medicine, Meram Medical Faculty, Konya University, Konya, Turkey.; 5 Basar Cander, Department of Emergency Medicine, Meram Medical Faculty, Konya University, Konya, Turkey.; 6 Mehmet Gul, Department of Emergency Medicine, Meram Medical Faculty, Konya University, Konya, Turkey.; 7 Sibel Doseyici, Department of Biochemistry, Meram Medical Faculty, Konya University, Konya, Turkey.; 8 Idris Mehmetoglu, Department of Biochemistry, Meram Medical Faculty, Konya University, Konya, Turkey.; 9 Tahir Kemal Sahin, Department of Public Health, Meram Medical Faculty, Konya University, Konya, Turkey.

**Keywords:** Ischemia, Ischemia-modified albumin, Emergency medicine, Acute coronary syndrome, Acute ischemic stroke

## Abstract

***Objective:*** To investigate diagnostic value of ischemia-modified albumin (IMA) levels in patients applying to emergency with symptoms of acute coronary syndrome (ACS) and acute ischemic stroke (AIS).

***Methods:*** Two patient groups (ACS and AIS) and a control group were constituted. The study was discontinued upon reaching 30 patients in each group. Following patient approval at the initial visit, a total of 10 ml venous blood sample was obtained from all patients with a high clinical suspicion of ACS and AIS. The Troponin I and the IMA levels were determined in the blood samples.

***Results:*** Statistically significant higher IMA values were determined in the patient groups compared to the control group (p<0.001 for both groups). No statistically significant correlation was found between the IMA and the Troponin I values in the ACS and the AIS groups (p>0.05 for both groups). The sensitivity of IMA was 83% and 87% for ACS and AIS, respectively. The specificity of IMA was 90% and 87% for ACS and AIS, respectively.

***Conclusion:*** The sensitivity and specificity values, determined according to the optimal cut-off values in the groups demonstrated that IMA could be a useful diagnostic marker in ACS and AIS patients.

## INTRODUCTION

Acute coronary syndrome (ACS) is an ischemic cardiac manifestation which may result in myocardial damage and necrosis parallel to prolonged duration of ischemia. In USA, the estimated yearly incidence of myocardial infarction is indicated as 610.000 new attacks and 325.000 recurrent attacks.^1^ Since ACS is a period of race against time, early diagnosis and treatment is crucial in terms of decreased mortality and morbidity**. **Currently, cardiac biochemical markers with high sensitivity and specificity are used in clinical practice; however, serum levels of these markers rise in a couple of hours after the attack, and negative results are found on presentation to the emergency department.^2^^,^^3^ Therefore, for the early diagnosis of ACS patients, studies related to newer markers such as heart-type fatty acid binding protein and N-terminal B-type natriuretic peptide are on-going.^4^^,^^5^

Acute ischemic stroke (AIS) is a state which results in brain cell death as the duration of ischemia is prolonged. In USA, 795.000 individuals are faced with new or recurrent stroke attacks each year and 87% of all stroke cases are ischemic.^1^ It is recommended that the time spent for imaging techniques should not delay the treatment, especially in patients who are candidates for intravenous fibrinolytic treatment.^6^ Regarding this issue, the diagnostic value of new biochemical markers like myelin basic protein, neuron specific enolase and B-type natriuretic peptide has been investigated.^7^^,^^8^

The ischemia-modified albumin (IMA) is a novel ischemia marker developed by quantifying the decrease in metal binding capacity.^9^ In recent years, a number of studies have been conducted on the use of IMA in the diagnosis of ACS and AIS with variable results.^10^^-^^12^ In this study, we investigated the IMA levels at the time of referral and the diagnostic value of these levels in patients presenting to the emergency department with symptoms of ACS and AIS.

## METHODS

Compliance with the Declaration of Helsinki was assured and the ethical guidelines were approved by the Institutional Local Ethical Committee. The study was conducted in the emergency department of a university hospital between March 2010-2011. A total of three groups, two patient groups (ACS and AIS) and a control group were constituted.

For the ACS group, patients over 18 years of age, presenting to the emergency department with symptoms of chest pain were enrolled and blood sampling was performed on presentation to the emergency department. The patients were monitored and the diagnosis of ACS was confirmed as per the following criteria in compliance with the guidelines^13^:

1. Clinical symptoms or new ECG abnormalities are consistent with ischemia and one biomarker is elevated above the 99th percentile of the upper reference limit.
2. ST-segment elevation or presumed new LBBB is characterized by ST-segment elevation in 2 or more contiguous leads.
3. Ischemic ST-segment depression >0.5 mm (0.05 mV) or dynamic T-wave inversion with pain or discomfort. Non-persistent or transient ST-segment elevation of ≥0.5 mm for <20 minutes.
4. Imaging evidence of new loss of viable myocardium or new regional wall motion abnormality.
5. Evidence of fresh thrombus by coronary angiography.

Patients with confirmed diagnosis of ACS were enrolled in the ACS group. For the AIS group, patients over 18 years of age, presenting to the emergency department with symptoms of acute focal or systemic stroke (e.g. alterations in consciousness, paralysis in extremities) with confirmed acute neurological deficit on the initial physical examination were initially enrolled and blood sampling was carried out. Patients with confirmed diagnosis of AIS by brain computerized tomography (CBT) and/or diffusion-weighted magnetic resonance (DWI) imaging were included in the AIS group. 

The control group comprised patients with no history of thromboembolism or immediate symptoms of a thromboembolic state on presentation to the emergency department with no findings related to hypoxic-ischemic disturbances. 

In all three groups, cases with serious trauma, acute-chronic liver and/or renal failure, coagulation disturbances and malignancy, pregnant women were excluded from the study. The study was terminated upon reaching 30 patients in each group. The demographic characteristics of patients, the diseases in the medical history, blood sampling time (time interval between the onset of symptoms and blood sampling was defined as “*blood sampling time*”), and the findings of the physical examination were recorded. 

A total of 10 ml venous blood sample was obtained from all patients with a high clinical suspicion of ACS and AIS. The blood sample was placed in two separate tubes, a gel vacutainer tube and a tube with EDTA. The sera were separated and kept at -80°C until the biochemical evaluations. For measurement of the IMA levels the spectrophotometric method described by Bar-Or was used and the results were reported as absorbance units (ABSU).^9^ The white blood cell (WBC) count, creatinine kinase (CK), mass creatinine kinase MB (mass CK-MB), Troponin I, C-reactive protein (CRP) were processed using routine kits.

The data were assessed using the SPSS, version 16.0. The Kruskal-Wallis variance analysis and the Mann-Whitney U test with Bonferroni correction were used in the comparison of non-normally distributed data. The Chi-square test was used for the categorical data. The Pearson correlation test was used in assessment of the correlation between variables. ROC curves were prepared to determine the diagnostic value of IMA levels in both disease groups. The sensitivity, specificity, positive predictive value (PPV), negative predictive value (NPV), accuracy rate (AR) and likelihood ratios (LRs) (with 95% confidence interval) were determined.

## RESULTS

A total of 90 patients, 30 cases in each group, were enrolled in this trial. The demographic characteristics and blood sampling times in the groups have been presented in Table-I. In the comparison of groups in terms of the demographic characteristics, the mean age in the AIS group was found to be significantly higher than both the control and the ACS groups (p=0.03). Twenty-six (86.7%) STEMI and 4 (13.3%) NSTEMI cases were found in the ACS group.

The serum biomarker levels in the groups have been presented in Table-II. The serum mass CK-MB, Troponin I and WBC count in the ACS group were significantly higher than those of the AIS group (p<0.001, p<0.001, p=0.04, respectively).

Statistically significant higher IMA values were determined in the patient groups compared to the control group (p<0.001 for both groups). Comparison of the IMA values in the ACS and the AIS groups did not reveal a statistically significant difference (p=0.26). No statistically significant correlation was found between the IMA and the Troponin I values in the ACS and the AIS groups (p>0.05 for both groups).

**Fig.1:**
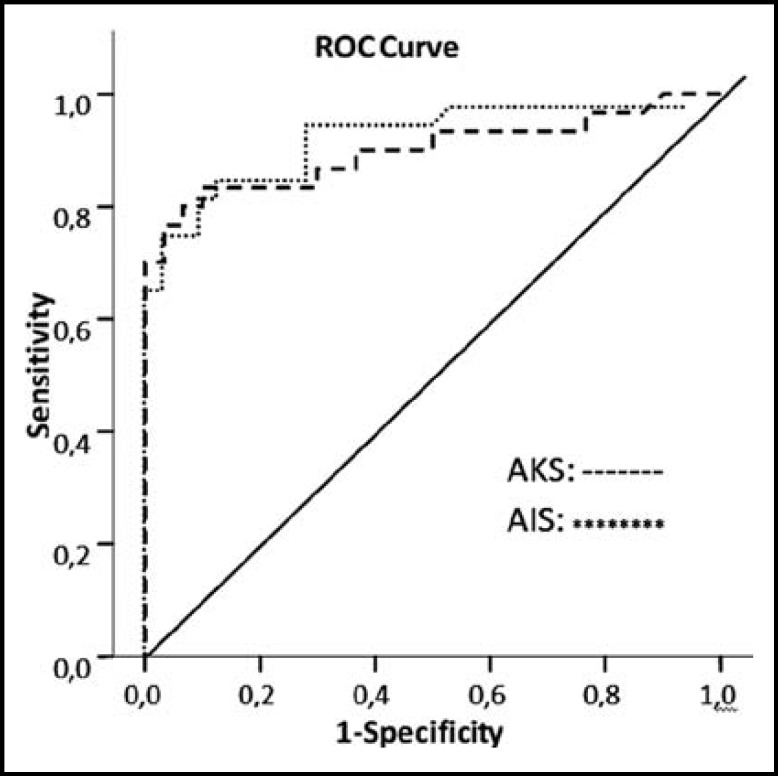
ROC curve for ACS and AIS patients in the IMA values

In the ACS and the AIS groups, the ROC curves were prepared for IMA levels and the area under the curve (AUC) was calculated. The ROC curves in each group have been shown in Fig.1. The sensitivity, specificity, PPV, NPV, AR and LR values with 95% confidence intervals, calculated for 0.85, 0.94 and 0.99 ABSU cut-off values in the ACS group and for 0.88, 0.93 ve 0.96 ABSU cut-off values in the AIS group have been demonstrated in Table-III.

**Table-I:** Demographic characteristics of the groups

	*ACS group* *(n=30)*	*AIS group* *(n=30)*	*Control group* *(n=30)*	*p value*
Age (mean±SD) as	57.2± 15.9	66.2±14.0	52.3±18.8	0.03
Gender (n (%))				
Female	10(%33.3)	18(%60.0)	16(%53.3)	0.16
Male	20(%66.7)	12 (%40.0)	14(%46.7)	
Medical history (n (%))				
HT	7(23.3%)	6(20.0%)	–	>0.05
DM	4(13.3%)	6(20.0%)	–	
CAD	8(26.7%)	7(23.3%)	–	
HL	5(16.7%)	5(16.7%)	–	
Others	6(20.0%)	6(20.0%)	–	
Blood sampling times (hours, the median)	6.2(1-96)	8.00(1-31)	–	0.38

**Table-II:** Biochemical measurement values of the groups

*Median±SD*	*ACS group * *(n=30)*	*AIS group* *(n=30)*	*Control group * *(n=30)*	*p value*
CK (u/lt)	479.6±416.3	207.0±7.1	Not tested	0.12
Mass CK-MB (g/ml)	71.5±79.5	2.5±1.8	Not tested	**<0.001**
Troponin I (ng/ml)	9.2±19.7	0.12±0.26	Not tested	**<0.001**
CRP (mg/l)	23.0±19.3	41.0±32.9	Not tested	0.06
WBC (K/ul)	12.0±3.6	8.7±3.3	Not tested	**0.04**
IMA (ABSU)	1.134±0.241	1.180±0.223	0.820±0.129	**<0.001**

**Table-III:** IMA values of the groups, different cut points as measured by sensitivity, specificity, PPV, NPV, AR ve LR values

	*AKS*			*AIS*		
AUC SE CI 95%	0.898 0.044 0.812-0.985			0.937 0.029 0.880-0.994		
Cut-off ABSU	0.85	0.94	0.99	0.88	0.93	0.96
Sensitivity % CI 95%	90 77-97	83 71-90	77 64-82	90 77-97	87 74-94	83 71-90
Specificity % CI 95%	60 47-67	90 77-97	93 81-99	70 57-77	87 74-94	90 77-97
PPV % CI 95%	69 59-75	89 76-97	92 77-99	75 64-81	87 74-94	89 76-96
NPV % CI 95%	86 67-96	84 73-91	80 69-85	88 71-97	87 74-94	84 73-91
AR % CI 95%	75 62-82	87 74-94	85 72-90	80 67-87	87 74-94	87 74-94
LR (+) CI 95%	2.25 1.44-2.97	8.33 3.11-29.83	11.50 3.33-67.22	3.00 1.78-4.26	6.50 2.83-16.34	8.33 3.11-29.83
LR (-) CI 95%	0.17 0.04-0.49	0.19 0.10-0.38	0.25 0.18-0.44	0.14 0.04-0.41	0.15 0.06-0.35	0.19 0.10-0.38

## DISCUSSION

Utilization of IMA levels in ACS patients which start to rise in the blood after a few minutes following ischemia were investigated in a number of trials in recent years. The results of trials indicate that the IMA levels show an early increase shortly after the onset of ischemia and maintain these high levels for 6-12 hours following ischemia.^3^^,^^14^^,^^15^ Therefore, IMA seems to be a useful marker to be used in patients presenting to the emergency department at the early and late stages following the onset of symptoms.

Liyan et al. performed coronary angiography on 113 patients presenting to the emergency department within 12 hours following the onset of an attack of chest pain; their results demonstrated that the albumin-cobalt binding capacity in ACS patients was significantly lower than that in patients presenting with non-cardiac chest pain.^14^ Chawla et al. determined the IMA levels in patients hospitalized in the coronary intensive care unit and in healthy individuals with no history of heart disease, and found significantly higher IMA levels in patients hospitalized in the coronary intensive care units compared to healthy individuals.^16^ In a trial conducted by Ozdem et al., the IMA levels were determined among patients evaluated in the emergency department with a pre-diagnosis of ACS and the serum IMA levels in the ACS group were reported to be significantly higher than the values in the healthy control group.^15^ In our study, the IMA levels in the ACS group were found to be significantly higher than the levels in the control group and these results were in compliance with the literature data.

In the trial of Ozdem et al., the sensitivity of serum IMA level was determined as 60.9%, specificity as 89.2%, PPV as 72.7% and the NPV value as 93%, for the diagnosis of ACS.^15^ In a trial performed by Anwaruddin et al., the authors reported that combined use of IMA and myocardial damage markers such as myoglobin and troponin T is a useful strategy in assessment of patients suspected to have a diagnosis of acute coronary ischemia and hence, concluded that IMA possesses a strong negative predictive value.^3^ In the trial conducted by Sinha et al., IMA, troponin T and ECG were measured in 208 patients presenting to the emergency department with the first attack of chest pain within three hours of the onset of symptoms. In the diagnosis of ACS patients with STEMI, NSTEMI and unstable angina, ECG as a single parameter displayed a sensitivity rate of 45% and troponin T showed a sensitivity rate of 20% as a single parameter; however, IMA showed a sensitivity rate of 82% as a single parameter, IMA and troponin T showed a rate of 90%, IMA and ECG as 92% and sensitivity of combination of IMA, ECG and troponin T was found as 95%.^2^ In a trial performed by Lee et al., conventional cardiac markers were negative in 13 of 129 patients with confirmed ACS, while an elevation was determined in IMA levels. The sensitivity was determined as 80–90%, NPV as 85–92% and specificity as 31–49%.^11^ In our study, the IMA cut-off value for ACS was determined as 0.94 (ABSU), while sensitivity was found as 83%, specificity as 90%, PPV as 89%, NPV as 84% and AR as 87%. The high sensitivity and specificity rates of IMA determined in our study indicate that this is a safe and promising method in terms of diagnosing ACS at the emergency department. On the other hand, advantages associated with IMA, namely the low cost, rapid results and easy implementation of the study protocol indicate that this test may be used as a powerful marker in clinical practice in the near future.

In various trials, free radicals have been shown to be increased in stroke cases, especially during reperfusion of ischemia.^17^ In the trial conducted by Abboud et al on four patient groups with intracerebral hemorrhage, infarction, transient ischemic attack (TIA) and epileptic seizures, significantly high IMA levels were determined in the patient groups compared to the control groups. Moreover, IMA levels in patients with TIA or seizures were significantly low compared to that of stroke patients. The sensitivity of IMA was determined as 57.8%, specificity as 81.3% and NPV as 21.7%.^12^ In a trial performed by Gunduz et al. on all stroke patients, the highest IMA levels were found in AIS patients. A statistically significant difference was found between the IMA levels of patients with AIS and subarachnoid hemorrhage. The sensitivity of IMA was determined as 86.8% and the specificity value was found as 60.5%.^18^ In our study, the IMA cut-off value for AIS was determined as 0.93 (ABSU), while sensitivity was specified as 86%, specificity as 87%, PPV as 87%, NPV as 87% and AR as 87%. In acute stroke cases where the diagnosis is primarily confirmed by radiological examinations, IMA seems to be a powerful candidate marker in the early diagnosis, due to high rates of sensitivity and specificity.

## CONCLUSION

The sensitivity, specificity, PPV and NPV values, determined according to the optimal cut-off values based on ROC curves in the groups demonstrated that IMA could be a useful diagnostic marker in ACS and AIS patients. These data should be supported by further comparative trials with other diagnostic markers conducted on larger patient populations.

## Authors Contribution:

Birsen Ertekin: Conceived the study design and Writing of manuscript.

Sedat Koçak: Designing the study.

Z.Defne Dündar: Supervised the study.

Sadık Girişgin: Data collection and financial resources.

Başar Cander: Data collection.

Mehmet Gül: Data Collection.

Sibel Döşeyici: Analysis of data.

İdris Mehmetoğlu: Literature Search.

Tahir Kemal Şahin: Critical Review.

## Notes

***Source of funding: ***Scientific Investigation Projects Directorate of Selcuk University, Konya, Turkey.
